# Optimized protocol for cryopreservation of human eggs improves developmental competence and implantation of resulting embryos

**DOI:** 10.1186/1757-2215-6-15

**Published:** 2013-02-13

**Authors:** Cassie T Wang, Lifeng Liang, Craig Witz, Dan Williams, Jason Griffith, Josh Skorupski, Ghassan Haddad, Jimmy Gill, Weihua Wang

**Affiliations:** 1 Houston Fertility Institute/New Houston Health, Houston, TX, USA; 2 Key laboratory of Major Obstetrics Diseases of Guangdong Province, the Third Hospital Affiliated to Guangzhou Medical University, Guangdong, China

**Keywords:** Blastocyst, Egg, Human, Implantation, Vitrification

## Abstract

**Background:**

Successful egg cryopreservation has many potential benefits to a variety of patients. However, a superior standard protocol describing all aspects of oocyte cryopreservation has not yet been identified. Oocyte cryopreservation is still a technical challenge for many infertility clinics. To maintain satisfactory clinical outcomes, there is a need to develop an easy to use, yet efficient laboratory protocol. The present study was designed to examine if human embryos resulting from eggs frozen with an optimized vitrification protocol have similar developmental competence as those from fresh eggs.

**Methods:**

Twenty recipients received donated eggs vitrified with a protocol in which short exposure time to the vitrification solution was used and 23 recipients received donated eggs and 6 patients had their own eggs vitrified with a modified protocol in which long exposure time to the vitrification solution was used. After warming, egg survival, fertilization, cleavage, blastocyst formation, clinical pregnancy and implantation rates were compared. The developmental competence of eggs vitrified with the optimized protocol was further compared with fresh eggs donated from the same donors.

**Results:**

There was no difference in the oocyte survival, fertilization, cleavage, clinical pregnancy or implantation rates between the short and long protocol groups. However, blastocyst formation rate was significantly (P < 0.001) higher in the long protocol group (50.8%) than that in short protocol group (26.5%), resulting in more blastocysts being transferred and frozen. When frozen eggs vitrified with long protocol and fresh eggs from the same donors (12) were compared in 39 recipients, no differences were observed in terms of fertilization (86.4 vs 80.1%), blastocyst formation (50.0 vs 59.2%), clinical pregnancy (63.2 vs 60.0%) and implantation (41.7 vs 44.7%) rates. Four out of 6 patients had ongoing pregnancy after transfer of embryos from their own frozen eggs with a 46.2% implantation rate.

**Conclusions:**

These results indicate that blastocyst development is an appropriate measure for egg survival after cryopreservation and frozen eggs have similar developmental potential as fresh eggs if they are frozen with an optimized method.

## Background

Successful egg cryopreservation has many potential benefits for patients. For example, young women can freeze their eggs for future use if they plan to have children later and young cancer patients can freeze their eggs before treatment so they can have children after recovery from cancer treatments that have ill-effects on existing oocytes [1]. Since the first birth of an in vitro fertilization (IVF) baby derived from fertilization of frozen-thawed eggs in humans [2], major progress has been made in this advanced assisted reproductive technology. There are mainly two laboratory methods to freeze human eggs: slow freezing [2] and vitrification [3]. When slow freezing was compared with vitrification, it was found that vitrified eggs had a better survival rate than slow freezing [4,5]. Several reports have indicated the inefficiency of the slow freezing technique that results in low survival and implantation rates [6-8]. Slow freezing can also cause spindle abnormalities [9] in human eggs. These factors have limited wide-spread clinical application of oocyte cryopreservation by slow freezing [8]. Although vitrification can also cause chromosome misalignment [10] in human oocytes, it has better results than slow freezing if appropriate protocols are used [11,12]. Vitrification is an alternative egg cryopreservation method that has been used recently by many laboratories [13-17]. Because vitrification can retain a high survival rate after thawing, it would appear that vitrification will become the main egg cryopreservation technology in the future.

More than 900 egg cryopreservation babies were born by 2009 and there was no apparent increase in congenital anomalies [18]. Recently, the American Society for Reproductive Medicine has opined that oocyte cryopreservation should no longer be considered experimental [19]. A few studies have reported high clinical pregnancy and live birth rates after transfer of the resulting embryos with vitrified/warmed human eggs [13,14,20-23]. It has also been reported that embryo development, clinical pregnancy and embryo implantation rates are similar between fresh and cryopreserved eggs in egg donation programs [20,24-26]. These successes have made it possible to establish egg banks for patients themselves and for patients with indications to receive egg donation. However, most of the reports were based on a Cryotop vitrification method [13,19-21,24-26] in which complicated laboratory procedures were used [27]. These procedures make this method difficult to be followed by laboratory technicians.

Many factors affect survival of eggs after vitrification [11,28-33], such as use of different cryoprotectant agents (CPAs) [28], the concentrations of CPAs [11], exposure time of eggs in the pre-vitrification and vitrification solutions [29] and temperature used for each step [30]. Different from embryo vitrification, it was found that gradual equilibration of eggs from low concentration of CPAs to high concentration of CPAs is one of the most important keys for successful vitrification of human eggs [6]. Taking into account of all these factors, various vitrification protocols have been developed [20,34,35]. These methods allow eggs to be equilibrated from low concentration of CPAs to high concentration of CPAs, which is better than direct exposure of eggs to high concentration of CPAs [36], in which eggs may be exposed to osmotic stress [37].

Unfortunately, a superior standard protocol describing all aspects of oocyte cryopreservation has not yet been identified. There are many nuances that remain between programs. Oocyte cryopreservation is still a technical challenge for many IVF clinics. To maintain satisfactory clinical outcomes, there is a need to develop an easy to use and efficient laboratory protocol. The purpose of the present study was to evaluate the effects of exposure time of oocytes in vitrification solution (VS) on subsequent oocyte survival, competence to develop to blastocysts, and successful implantation after transfer. To simplify the data analysis, this retrospective study was limited to oocytes from anonymous donors and women under 40 years of age who wished to limit the number of oocytes inseminated, and cases in which sperm was not available on the day of oocyte retrieval.

## Methods

### Ethics

Patients undergoing IVF, egg cryopreservation, and egg donation signed written consents for all kinds of laboratory and clinical procedures. All egg donors were anonymous in the present study. The data was collected from the medical records at the clinic between January 2011-August 2012, and the study was approved by the institutional research committee at Houston Fertility Institute, and the Medical Ethics Committee and Institutional Review Board of the third Affiliated Hospital of Guangzhou Medical University.

### Patient preparation for egg retrieval

Volunteer oocyte donors and patients underwent controlled ovarian hyperstimulation as described below. Oocytes were inseminated if recipients were prepared for subsequent embryo transfer. Supernumerary oocytes were cryopreserved and stored in an egg bank. These oocytes were subsequently thawed and inseminated if recipients requested. In some cases of donation, all of the oocytes were placed into the egg bank. In addition, some patients had their oocytes cryopreserved due to failed collection of semen on the day for egg retrieval.

Patients, donors and women cryopreserving their own oocytes, were stimulated with a combination of Follistim (Organo Inc, Roseland NJ, USA), Gonal-F (EMD Serono, Rockland MA, USA), Menopur (Ferring Pharmaceuticals, Parsippany NJ, USA) and/or Bravella (Ferring Pharmaceuticals) beginning 2–3 days after the onset of menses. The initial starting total dose was 150–375 IU and was adjusted subsequently as the stimulation progressed. To prevent an LH surge, a GnRH antagonist, Ganirelix or Cetrorelix (Organo Inc.), was given when the leading follicle was 13–14 mm or when the estradiol level was 400 pg/ml. Human chorionic gonadotropin (hCG), Ovidrel (Serono USA), or a GnRH agonist, leuprolide acetate (Teva North America, North Wales PA, USA), was injected to induce final oocyte maturation when at least two dominant follicles reached a diameter of >18 mm. Eggs were retrieved under IV sedation via transvaginal ultrasound between 35–37 hours after hCG administration. Oocytes were inseminated using intracytoplasmic sperm injection (ICSI).

### Egg vitrification

Oocytes were cultured for 3–5 hours before removing the surrounding cumulus cells in a HEPES-buffered global medium (IVFonline, CT, USA) containing 40 iu hyaluronidase. Only mature (metaphase II) oocytes were vitrified. Vitrification was initially performed based on a protocol in which eggs were exposed to the VS for 45 seconds (short protocol) and then was modified by increasing the time in the VS to 90 seconds (long protocol). Closed vitrification straws and Irvine vitrification kits (Irvine Scientific, Irvine CA USA) were used in this study.

**Figure 1:**
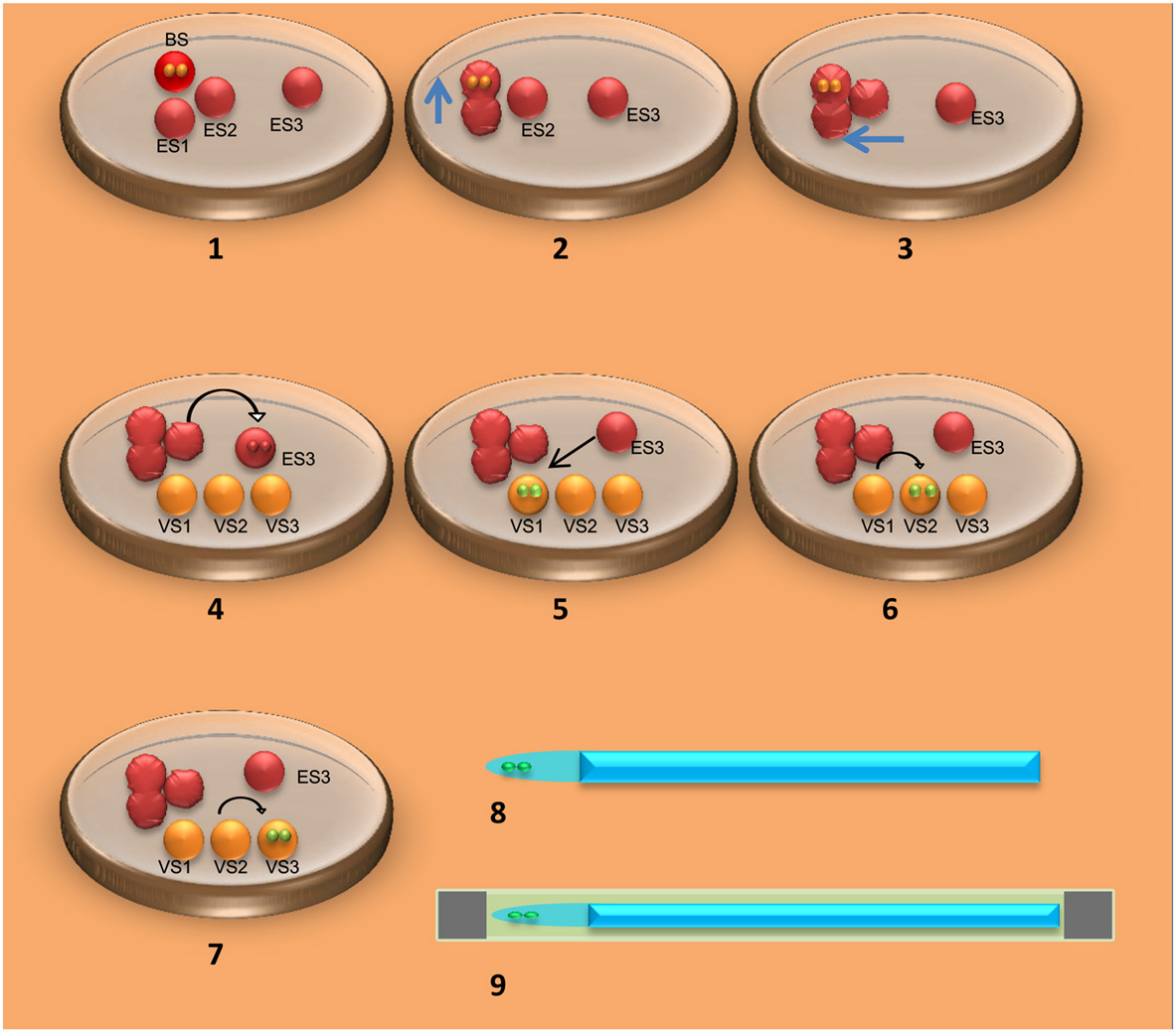
**Schematic diagram for egg vitrification used in the study.** A 20 μl of basic solution (BS) drop without CPAs and three 20 μl drops of equilibration solution (ES) are made in a cover of a 60 mm culture dish (**1**). Eggs are placed in the BS for 1 minute and then merged to 1 drop of ES using a transfer pipette for 2 minutes (**2**). The second ES drop is merged to the solution using the same transfer pipette for 2 minutes (**3**). Eggs are transferred from the merged solution to the third drop of ES for 5 minutes (**4**). When the eggs are in the third drop of ES, three 20 μl drops of vitrification solution (VS) are made in the same culture dish (**4**). Eggs are transferred to VS drop 1 (**5**), then to VS drop 2 (**6**), and finally to VS drop 3 (**7**). Eggs are remained in each drop for 10–20 seconds. The equilibrated eggs are finally loaded to the tip of the vitrification straw before the time reaches 90 seconds (**8**). Vitrification straw is inserted to cooled protective straw inside liquid nitrogen when the time reaches 90 seconds and the top end of the protective straw is sealed (**9**).

All procedures were performed at room temperature (22-25°C). Briefly, as shown in Figure 1, eggs were equilibrated in 20 μl drop of basic solution (BS) for 1 minute before the drop was merged to a 20 μl drop of the equilibration solution (ES) containing 7.5% (v/v) ethylene glycol (EG) and 7.5% (v/v) dimethylsulphoxide (DMSO) for 2 minutes, and then a second 20 μl drop of ES was merged for another 2 minutes. The eggs were then placed into a new 20 μl drop of ES for 5 minutes. After equilibration, the eggs were transferred into three 20 μl drops (10~20 seconds in each drop) of VS that was composed of 15% (v/v) EG, 15% (v/v) DMSO and 0.5 M sucrose and then loaded onto half-cutting 0.25 ml straws. The straws were inserted into 0.5 ml protective straws (one end was sealed) inside liquid nitrogen for cryopreservation and then the top opening of the protective straw was sealed (Figure 1). The time from the eggs being placed in the first drop of VS to the vitrification straw being placed in liquid nitrogen was 45 seconds in Group A (short protocol) and 90 seconds in Group B (long protocol). Two to three eggs were vitrified in one straw and then the frozen eggs were stored in the liquid nitrogen until warming for insemination.

### Egg warming and insemination

Egg warming was based on the procedures previously reported [20]. Briefly, straws were removed from liquid nitrogen and the tips of the straws with eggs were quickly placed in 1 ml 1.0 M sucrose that had been warmed at 37°C in an organ culture dish. After 1 minute in the 1.0 M sucrose solution, eggs were transferred to 1 ml 0.5 M sucrose for 3 minutes and then to 1 ml BS for 10 minutes. These procedures were performed at room temperature (22-25°C). After warming, eggs were washed with Global medium supplemented with 10% serum protein substitute (SPS, IVFonline CT, USA) and then cultured in the same medium for 2–3 hours before ICSI. Egg survival status was evaluated based on morphology immediately after completion of the warming procedures and ICSI was performed by experienced embryologists whose skill levels have been verified by their high fertilization rates. All eggs were cultured in Global medium supplemented with 10% SPS after ICSI.

### Embryo culture and transfer

Fertilization was examined 16–18 hours after ICSI and zygotes were cultured in Global medium supplemented with 10% SPS at 37°C in a humidified atmosphere of 5.5% CO_2_, 5% O_2_ and balanced nitrogen until day 6 after inseminations. On Day 5, embryo development was evaluated and the best 1–2 embryos depending on embryo quality were transferred.

All patients for embryo transfer received estradiol orally and transvaginally. Intramuscular administration of progesterone oil was initiated after about 14 days of estradiol treatment. Endometrium thickness was measured on the day of progesterone administration. Embryo transfer occurred on the sixth or seventh day of progesterone administration and progesterone was continued until the first serum β-hCG test two weeks after transfer.

### Pregnancy diagnosis

Fourteen days after embryo transfer, a serum β-hCG was checked. When the β-hCG showed > 5 mIU/ml, the patients were regarded as a biochemical pregnancy. Four weeks after embryo transfer, when a gestational sac with fetal heartbeat was seen in the uterus under ultrasonography, patients were diagnosed as having a clinical pregnancy.

### Statistical analysis

Interval and ratio data were analyzed with one-way ANOVA and differences in categorical data between groups were analyzed by Chi-square. A p value of less than 0.05 was considered to be statistically different.

## Results

### Long time exposure of eggs in VS is better than short time exposure

As shown in Table 1, in the present study, twenty cycles used 151 eggs (7.6±1.5 per cycle) vitrified with the short protocol and 23 cycles used 183 eggs (7.9±0.9 per cycle) vitrified with the long protocol. There were no differences between the two groups in terms of average age of donors and recipients, egg survival rates after warming, normal fertilization rates, percentages of cleaved embryos and endometrium thickness in the recipients. However, blastocyst development rate was significantly lower (P< 0.001) in the short protocol group (26.5%) than that in the long protocol group (50.8%). There were no statistical differences in the clinical pregnancy, ongoing pregnancy and embryo implantation rates although higher rates were found in the cycles using eggs vitrified with the long protocol than those with the short protocol.

**Table 1:** Clinical summary of warming cycles of vitrified donor eggs*

	**Group A**	**Group B**	**P Value**
No. of cycles	20	23	NA
Mean age of donors	28.2±1.7	25.6±1.8	>0.05
Mean age of recipients	42.4±6.0	40.2±4.2	>0.05
Endometrium thickness (mm)	9.7±1.2	9.4±1.7	>0.05
No. of eggs/warming cycle	7.6±1.5	7.9±0.9	>0.05
Total No. of eggs warmed	151	183	NA
No. of eggs survived (%)	142 (94.0)	174 (95.1)	>0.05
No. of eggs fertilized (%)	114 (80.3)	145 (83.3)	>0.05
No. of zygotes cleaved (%)	98 (86.0)	132 (91.0)	>0.05
No. of blastocysts (%)	26 (26.5)	67 (50.8)	<0.001
No. of clinical pregnancy (%)	9 (45.0)	15 (65.2)	>0.05
No. of ongoing pregnancy or delivery (%)	8 (40.0)	13 (56.5)	>0.05
Total No. of embryos transferred	39	44	NA
Mean No. of embryos transferred	1.95±0.51	1.91±0.29	>0.05
No. of embryos implanted (%)	12 (30.8)	20 (45.5)	>0.05

* Eggs from different donors were used in Groups A and B.

### More patients had blastocyst transfer and cryopreservation if the eggs were frozen with the long protocol

When we further analyzed the detailed embryo development, as shown in Table 2, we found that if the short protocol was used, 60% of the recipients had at least one available blastocyst for transfer, and the pregnancy (58.3%) and implantation (41.6%) rates with blastocyst transfer were higher than those (25.0% pregnancy rate and 13.3% implantation rate) without blastocyst transfer that accounted for 40% of the cycles. Only 25% cycles had spare blastocysts for cryopreservation. However, when the long protocol was used, 87% of the recipients had available blastocysts for transfer and 65.2% had spare blastocysts for cryopreservation. The pregnancy (70.0%) and implantation (50.0%) rates were also higher in the patients with blastocyst transfer than those (33.3% pregnancy rate and 16.7% implantation rate) in the patients without a blastocyst transfer (Table 2).

**Table 2:** Summary of warming cycles of frozen donor eggs with or without blastocyst transfer^*^

	**Group A**		**Group B**	
Embryos for transfer	with blastocyst(s)	without blastocyst	with blastocyst(s)	without blastocyst
No. of transfers	12	8	20	3
% in the group	60	40	87	13
Mean age of recipients	44.9±5.1	38.5±5.3	40.6±4.1	37.7±4.9
Total No. of embryos transferred	24	15	38	6
Mean No. of embryos transferred	2.0±0.0	1.9±0.8	1.9±0.3	2.0±0.0
No. of clinical pregnancy (%)	7 (58.3)	2 (25.0)	14 (70.0)	1 (33.3)
No. of embryos implanted (%)	10 (41.6)	2 (13.3)	19 (50.0)	1 (16.7)

*Data from same patients showed in Table 1 was analyzed.

### Frozen eggs and fresh eggs had similar blastocyst development and implantation rates

In the present study, 39 recipients received either fresh eggs or frozen eggs (vitrified with the long protocol) from 12 egg donors with an average of 3.2 recipients per donor. When we further analyzed the data between frozen eggs and fresh eggs, we did not find any difference in recipient’s ages, fertilization rates, blastocyst formation rates, clinical pregnancy rates, ongoing pregnancy rates and implantation rates between two groups except that a higher (P <0.05) cleavage rate (99.5%) was observed in the fresh eggs than that (92.6%) in the frozen eggs (Table 3).

**Table 3:** Summary of cycles of frozen eggs vs fresh eggs from 12 same donors

	Frozen eggs*	**Fresh eggs**	**P Value**
No. of cycles	19	20	NA
Mean age of recipients	40.1±4.4	42.1±5.5	>0.05
No. of eggs/warming cycle	7.7±0.7	13.2±7.5	>0.05
Total No. of eggs warmed or used	147	263	NA
No. of eggs survived (%)	140 (95.2)	NA	NA
No. of eggs fertilized (%)	121 (86.4)	212 (80.6)	>0.05
No. of zygotes cleaved (%)	112 (92.6)	211 (99.5)	<0.05
No. of blastocysts (%)	56 (50.0)	125 (59.2)	>0.05
No. of clinical pregnancy (%)	12 (63.2)	12 (60.0)	>0.05
No. of ongoing pregnancy or delivery (%)	10 (52.6)	11 (55.0)	>0.05
Total No. of embryos transferred	36	38	NA
Mean No. of embryos transferred	1.9±0.3	1.9±0.6	>0.05
No. of embryos implanted (%)	15 (41.7)	17 (44.7)	>0.05

*All eggs were vitrified with the long protocol.

**Figure 2:**
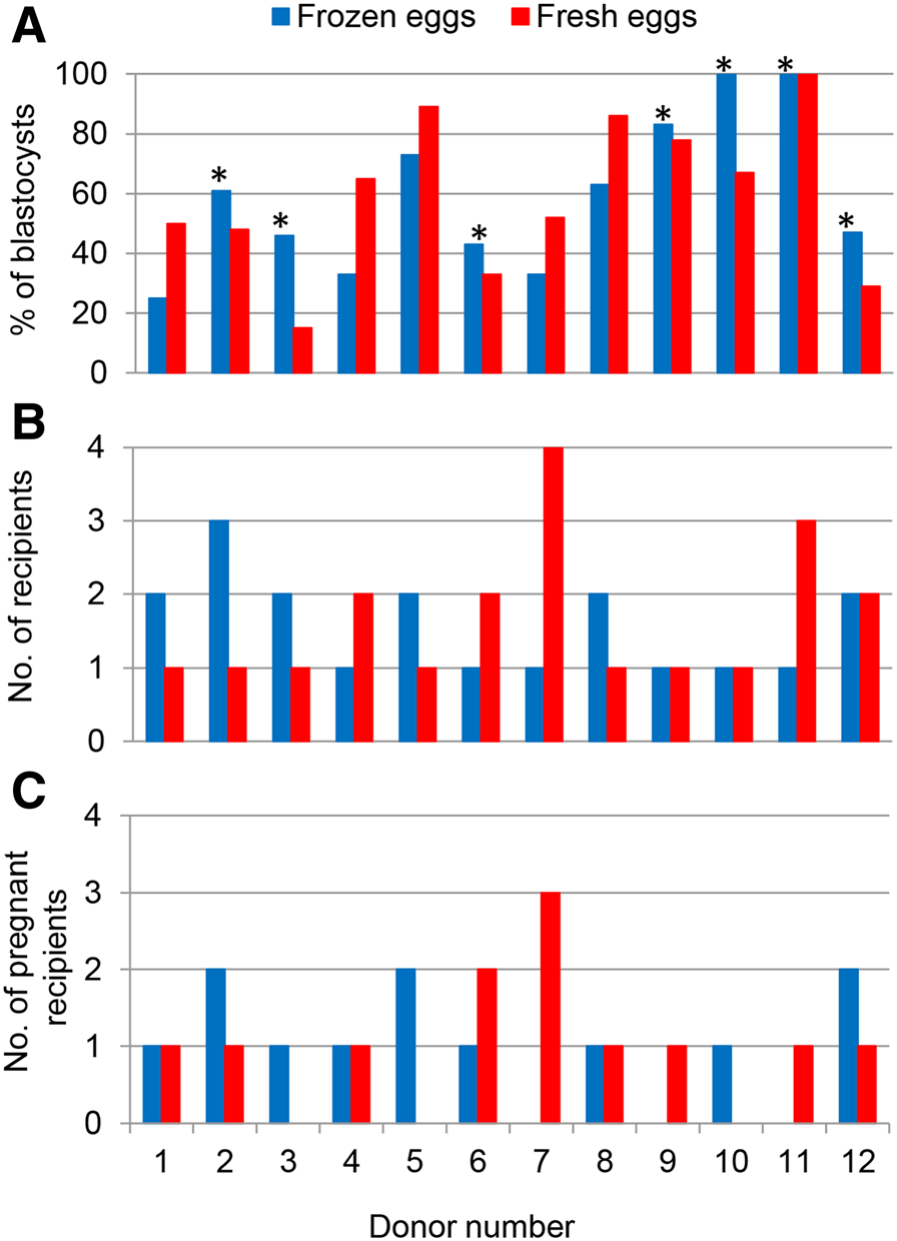
**Direct comparison of fresh eggs and frozen eggs from 12 individual donors.** There are no statistical significant differences in the blastocyst development among 12 donors between fresh and frozen eggs, in which 7 donors (marked by stars) had more or equal blastocyst development rates as compared with their fresh eggs (**A**). Up to 5 recipients received eggs from the same donor and the numbers of cycles with frozen and fresh eggs are shown in **B**, and the numbers of pregnant recipients from each donor are shown in **C**. Long protocol was used to freeze the eggs and data was from the same patients as shown in Table 3.

Because the blastocyst development is one of the most important indicators for subsequent embryo implantation, we further analyzed the 12 donors to evaluate if there is any difference between fresh and frozen eggs in the same individual donors. As shown in Figure 2, we found that blastocyst development rates between fresh and frozen eggs did not show statistical differences in all 12 donors.

### High implantation rate of the embryos resulting from frozen patients own eggs

The long protocol was used to freeze eggs from patients in the present study due to the lack of sperm or failed semen collection at the egg retrieval day. The outcome was summarized in Table 4. We obtained a 48.4% blastocyst rate in the present study and all patients had blastocysts for transfer. As a result, 5/6 patients had clinical pregnancy and 4 patients had ongoing pregnancy with a 46.2% implantation rate.

**Table 4:** Summary of egg vitrification and warming in the non-donor patients *

**Case**	**Age**	**No. of oocytes survived**	**No. of oocytes fertilized**	**No. of zygotes cleaved**	**No. of embryos transferred**	**Clinical pregnancy**	**Ongoing pregnancy**	**No. of embryos implanted**
1	39	6/7	6	4	Blast x2	No	No	0
2	30	4/4	3	3	Blast x2	Yes	Yes	1
3	39	9/10	6	5	Blast x2 (2)**	Yes	Yes	1
4	34	10/10	8	8	Blast x2	Yes	No	1
5	36	4/4	4	4	Blast x2	Yes	Yes	1
6	29	8/8	7	7	Blast x2 (1)**	Yes	Yes	2
Total		41/43	34	31	13 (15)***	5	4	6
%		95.3	82.9	91.2	48.4	83.3	66.7	46.2

*Eggs were vitrified with the long protocol.

**Numbers in the parentheses are the spare blastocysts for cryopreservation.

***Number in the parentheses is the total number of blastocysts (transferred and cryopreserved).

## Discussion

In the present study, we demonstrated that by optimizing a vitrification protocol, cryopreserved and fresh human eggs have similar developmental competence with respect to blastocyst formation. Transfer of embryos resulting from frozen eggs produced similar clinical pregnancy and implantation rates as fresh eggs. Our results indicate that post warming survival, fertilization and cleavage are not good indicators for frozen egg survival, but blastocyst development is a better indicator for egg survival after freezing/thawing. These results suggest that frozen egg banks can be established after considering the efficiency of production of high quality embryos and ongoing pregnancy.

Gradual exposure of eggs to ES during equilibration is important for egg vitrification. A few years ago, Kuwayama developed a gradual exposure method for egg vitrification using a specifically designed culture dish and reported a high post warming survival rate [27]. However, a special culture dish is required for vitrification of eggs with Kuwayama’s method, and also a particular handling is necessary during vitrification, which makes it difficult to be followed by others. In some other reports, oocytes were vitrified after being equilibrated in different concentrations of ES made in-house [22,35]. However, such multiple step solutions are not commercially available. In the protocol designed in the present study, eggs were first placed in a drop of BS and then merged to an adjacent drop of ES twice, finally placed in a new drop of ES. Through this method, the eggs can also be equilibrated from low concentration of CPAs to high concentration CPAs with the standard commercial embryo cryopreservation solutions.

Exposure time of eggs in different ES and VS before vitrification varies among protocols. These steps appear to be key components for successful cryopreservation of eggs. Some researchers have used a maximum of 15 minutes in the ES depending on egg shape recovery [20,34,38]. It may be difficult for embryologists to evaluate the optimal equilibration status of an egg. To simplify the protocol, a fixed time in each step may be necessary for all laboratory technicians to follow. Therefore, in the present study, we used a fixed time for a total of 9 minutes in the ES and found that it was appropriate for vitrification of human eggs if the time in VS was appropriate. This time period is obviously shorter than other reports, which may be better for the egg’s viability by reducing time of exposure eggs to the toxic CPAs. We found that this method is very practical and easy to use and learn in the laboratory. Importantly, this method yielded high egg survival rates and favorable clinical outcomes.

Another major key for egg vitrification is the exposure time of eggs in the VS. It was reported that the short time was essential for vitrification of human eggs and embryos, because high concentrations of CPAs were added in the VS [39]. Many early reports suggested that the time should be less than 10 seconds [40]. However, based on the studies in the mouse and human, short exposure of eggs to VS was unfavorable to egg survival and their competence to develop to blastocysts after vitrification [23,29,41]. In some early reports, it was found that vitrified mouse and human oocytes could survive and form embryos by using brief exposure to VS, but the survival rates and blastocyst formation rates were quite low [41-43]. In the present study, we used two different exposure times for vitrification, one was 45 seconds and another was 90 seconds. We found that post warming survival, fertilization and cleavage rates were not statistically different between both groups. However blastocyst development rates were significantly lower in the short protocol group. This suggests that intracellular water may not be completely replaced by CPAs in the eggs exposed to VS for a shorter time. This could result in damage to some organelles inside the eggs, which escape detection via microscopic evaluation of general oocyte morphology in the present study.

Outcomes from vitrified donor eggs and sibling fresh donor eggs derived from the same stimulation cycles were compared in the present study. There were no significant differences in fertilization rates and blastocyst development rates except that the cleavage rate was lower in the frozen eggs than that in fresh eggs. Our results are in contradistinction to a previous study that indicated that embryo quality was affected during the procedure of cryopreservation when frozen-thawed oocytes were compared with fresh oocytes [24] but comparable to those reported by others [20,25,44]. Our results indicate that not only fertilization and blastocyst development rates, but also the implantation rates were similar between frozen and fresh eggs.

Creation of cryo-stored oocyte banks for oocyte donation has been advocated in recent years. Accumulated data from different clinics indicate that oocyte cryo-banking using vitrification represents advancement in oocyte donation [20,25,44]. Egg banks have the potential to lower cost and also overcome the obstacle of synchronization of the recipients’ cycles with that of the donor [20,21,23,26,45,46]. The sharing of cryo-donated oocytes to several infertile recipients is a means of reducing waiting time and cost for recipients. Most importantly, the use of frozen donated eggs can produce satisfactory pregnancy rates [14,20,25,47]. In the present study, we also obtained similar rates of blastocyst development, embryo implantation and clinical pregnancy by using frozen and fresh oocytes obtained from the same donors. These results indicate that our optimized protocol for egg cryopreservation does not disturb eggs’ structure and function during freezing and thawing.

Most studies on egg cryopreservation were focused on donated eggs from young women [20,21,23,25,26,46]. It would be possible that eggs from young women or egg donors can tolerate cryo-injuring better than eggs from women with advanced maternal age or infertile women. In the present study, with limited data, we observed high survival, fertilization, and embryo implantation rates when the eggs from infertility patients were vitrified/warmed. These preliminary results are encouraging and suggest that the protocol used in the present study is not only appropriate for eggs from young donors, but also for eggs from infertile patients.

Although the survival rate of egg vitrification can exceed 90% after warming, some eggs may not be able to develop further after insemination. The survival rate immediately after egg warming may not be a good indicator for egg competence after cryopreservation. For example, in the present study, we found that the egg survival rate was high but blastocyst development rate was low if the short protocol was used. These results indicate that blastocyst development would be a better indicator for egg cryopreservation and should be used as the primary indicator of egg survival or egg competence after cryopreservation.

## Conclusions

Our results indicate that human eggs can be successfully vitrified with an easy to use protocol, and frozen human eggs have a similar developmental competence to fresh eggs with respect to the rates of blastocyst formation and embryo implantation. Our results also indicate that blastocyst formation and embryo implantation are two key indicators to assess the success of an egg vitrification protocol.

## Abbreviations

BS: Basic solution; CPAs: Cryoprotectant agents; DMSO: Dimethylsulphoxide; EG: Ethylene glycol; ES: Equilibration solution; hCG: Human chorionic gonadotropin; ICSI: Intracytoplasmic sperm injection; IVF: In vitro fertilization; LH: Luteinizing hormone; VS: Vitrification solution; SPS: Serum protein substitute.

## Competing interest

We declare that we have no conflict of interest.

## Authors’ contributions

CTW and WW carried out study design, egg freezing and thawing. CW, DW, JGr, JS, GH and JG did clinical patient evaluation, and procedures for egg retrieval and embryo transfer; CTW, LL and WW did statistical analysis and drafted manuscript. All authors read and approved the final manuscript.
